# Cellular Markers of Active Disease and Cure in Different Forms of *Leishmania infantum*-Induced Disease

**DOI:** 10.3389/fcimb.2018.00381

**Published:** 2018-11-13

**Authors:** Laura Botana, Belén Matía, Juan V. San Martin, Alberto Romero-Maté, Alicia Castro, Laura Molina, Laura Fernandez, Ana Ibarra-Meneses, Marta Aguado, Carmen Sánchez, Luis Horrillo, Carmen Chicharro, Javier Nieto, Sheila Ortega, José Manuel Ruiz-Giardin, Eugenia Carrillo, Javier Moreno

**Affiliations:** ^1^WHO Collaborating Centre for Leishmaniasis, National Centre for Microbiology, Instituto de Salud Carlos III, Madrid, Spain; ^2^Hospital Universitario de Fuenlabrada, Fuenlabrada, Madrid, Spain; ^3^Programa de Doctorado en Ciencias de la Salud, Escuela Internacional de Doctorado, Universidad Rey Juan Carlos, Mostoles, Spain

**Keywords:** visceral leishmaniasis, cutaneous leishmaniasis, lymphadenopathic leishmaniasis, mucosal leishmaniasis, biomarker, cure, cell proliferation assay, IFN-γ

## Abstract

Increased numbers of peripheral blood mononucleocytes (PBMC) and increased IFN-γ secretion following *in vitro* challenge of blood samples with soluble *Leishmania* antigen (SLA), have been proposed as biomarkers of specific cell-mediated immunity, indicating that treatment of visceral leishmaniasis (VL) has been successful. However, *Leishmania infantum* infection may manifest as cutaneous leishmaniasis (CL), and less commonly as localized leishmanial lymphadenopathy (LLL) or mucosal leishmaniasis (ML). The present work examines the value of these biomarkers as indicators of cured leishmaniasis presenting in these different forms. Blood samples were collected before and after treatment from patients living in Fuenlabrada (Madrid, Spain), an *L. infantum-*endemic area recently the center of a leishmaniasis outbreak. All samples were subjected to *Leishmania*-specific PCR, serological tests (IFAT and rK39-ICT), and the SLA-cell proliferation assay (SLA-CPA), recording PBMC proliferation and the associated changes in IFN-γ production. Differences in the results recorded for the active and cured conditions were only significant for VL. PCR returned positive results in 67% of patients with active VL and in 3% of those with cured leishmaniasis. Similarly, rK39-ICT returned a positive result in 77% of active VL samples *vs*. 52% in cured VL samples, and IFAT in 90% *vs*. 56%; in the SLA-CPA, PBMC proliferation was seen in 16% *vs*. 90%, and an associated increase in IFN-γ production of 14 and 84%, respectively. The present findings reinforce the idea that PBMC proliferation and increased IFN-γ production in SLA-stimulated PBMC provide biomarkers of clinical cure in VL. Other tests are urgently needed to distinguish between the cured and active forms of the other types of clinical leishmaniasis caused by *L. infantum*.

## Introduction

### A wide spectrum of clinical forms produced by *L. infantum*

Leishmaniasis is a neglected, vector-borne disease with high morbidity caused by protozoan pathogens of the genus *Leishmania*. Over 350 million people in some 98 countries are at risk, and an estimated 1.3 million new cases of leishmaniasis are reported every year (WHO, 2010).

Infection with *Leishmania infantum* is manifested in different clinical forms. The most severe is visceral leishmaniasis (VL), in which the parasite is systemically disseminated; it is fatal if untreated. Cutaneous leishmaniasis (CL) is a benign form caused by the multiplication of *Leishmania* in the skin; these infections usually clear up spontaneously. Both these clinical forms are seen in Mediterranean countries. During the *L. infantum*-caused outbreak of leishmaniasis in Fuenlabrada (Madrid, Spain), VL was seen in about a third of people with clinical infection, and CL in most of the remainder (Suárez Rodríguez et al., 2012; Arce et al., 2013); a small number presented with mucosal leishmaniasis (ML) and leishmanial localized lymphadenopathy (LLL). LLL is characterized by long term lymphadenopathy with neither fever nor any other systemic symptom (Horrillo et al., 2015). These differences in the clinical presentation of the disease seem mainly attributable to the patient's immune status. Certainly, the molecular typing of *L. infantum* isolates from patients in Fuenlabrada revealed no association between genotype and disease type (Chicharro et al., 2013).

### Biomarkers are needed that can identify cured patients following treatment for leishmaniasis

Treatment for leishmaniasis is usually effective, but relapses are common, especially in immunosuppressed patients (van Griensven et al., 2014). Confirmation of a final cure in both CL and VL is still based on clinical features after follow-up periods of 6 months; relapses commonly occur long after treatment has ended (Rijal et al., 2013). In immunocompetent patients with VL, a successful response to therapy depends on the activation of a Th1 subset of CD4+ *Leishmania*-specific T cells, and the production of IFN-γ, which induces macrophage leishmanicidal activity (Kemp et al., 1993). Indeed, the proliferation of blood mononucleocytes (PBMC) after challenge with soluble leishmanial antigen (the soluble *Leishmania* antigen cell proliferation assay [SLA-CPA]) (Singh and Sundar, 2014; Carrillo et al., 2015), and increased IFN-γ secretion by these cells (Hailu et al., 2004; Kumar et al., 2014), provide *in vitro* markers that might be used to assess early response to treatment. In fact, PBMC proliferation has been shown a useful indicator of the existence of *Leishmania*-specific T cell memory clones in HIV+ patients, sufficient to keep the parasitic infection under control and avoid relapsing VL (Castro et al., 2016). The value of IFN-γ in monitoring the cellular immune response has previously been reported in VL caused by *L. infantum* (Cillari et al., 1995; Adem et al., 2016; Ibarra-Meneses et al., 2017).

The present work examines the value of these biomarkers and others as indicators of cured leishmaniasis presenting in its different forms; their sensitivity and specificity have been said to vary across the different manifestations of the disease (Kip et al., 2015). The results confirm that PBMC proliferation and increased IFN-γ production following the challenge of blood samples with SLA, can be used to identify patients cured of VL. However, new biomarkers are required that are able to indicate the same in the other forms of *L. infantum*-induced disease.

## Materials and methods

### Study group, blood samples, and tests performed

The study subjects were 141 adult patients from Fuenlabrada (Madrid, Spain). All were diagnosed at the Hospital Universitario de Fuenlabrada between January 2013 and June 2015. Blood samples were collected during the period of active disease and after apparent cure. Active VL, CL, and ML was defined according to WHO definitions (WHO, 2010). LLL was defined as isolated adenopathy with no other systemic symptom (Ignatius et al., 2011; Horrillo et al., 2015). Blood samples were obtained from 33 patients with active VL, 27 from those with active CL, 6 from those with active LLL, and 2 from those with active ML. Post-supposed-cure blood samples were available from 61 patients originally diagnosed with active VL, 41 with CL, 21 with LLL, and 3 with ML, with cure defined as being free of leishmaniasis symptoms 6 months after the end of treatment (WHO, 2010). None of the cured patients has relapsed after 2 further years of follow-up.

All samples were analyzed by *Leishmania*-specific nested PCR to detect leishmanial DNA. *Leishmania*-specific antibodies were determined in plasma using the rK39 immunochromatographic test (rK39-ICT) and immunofluorescent antibody test (IFAT). PBMC proliferation after stimulation with SLA was measured (SLA-CPA assay), and the accompanying production of IFN-γ recorded.

### DNA extraction and nested PCR

Genomic DNA was extracted from 100 μl of peripheral blood using the QIAamp DNA Mini Kit (Qiagen, Germany) following the manufacturer's recommendations, and eluted in a final volume of 200 μl of PCR-grade water. The extracts were stored at 4°C until PCR analysis (maximum of 3 days). *Leishmania* DNA was detected by nested PCR (LnPCR), targeting the small subunit ribosomal ribonucleic acid gene SSU-rRNA (18S RNA). A first amplification step was performed using primers R221 and R332 (van Eys et al., 1992); the PCR product was then tested in a subsequent amplification step with primers R233 and R333, as described by Cruz et al. (2002).

### Immunofluorescent antibody test

2 × 10^5^ *L. infantum* promastigotes (JPC strain MCAN/ES/ 98/LLM-722) in PBS were fixed to glass slides. Two-fold serial dilutions of plasma—from 1/20 to 1/640 in PBS—were then added to separate preparations. The total IgG concentration was then determined by adding fluorescein isothiocyanate-conjugated goat anti-human IgG (Fluoline G) (Bio-Mérieux, France) diluted 1/200. The threshold titre for positivity was set at the 1/80 plasma dilution level.

### rK39 immunochromatographic test

Antibody detection was performed using the dipstick format Kalazar Detect Rapid Test (InBIOS International, Seattle, WA) according to the manufacturer's instructions.

### Preparation of soluble *L. infantum* antigen for SLA-CPA

*L. infantum* antigen extract was prepared from stationary phase promastigote cultures (JPC strain, MCAN/ES/98/LLM-722) as previously described (Carrillo et al., 2015). Briefly, parasites resuspended in lysis buffer (50 mM Tris/5 mM EDTA/HCl, pH 7) were subjected to three rapid freeze/thaw cycles followed by three 20 s 40 W pulses with a sonicator. These samples were then subjected to two consecutive rounds of centrifugation at 27,000 *g* and 4°C for 20 min. The supernatants were then collected, aliquoted, and stored at −80°C until use. Protein quantification was performed using the Bradford method employing the Bio-Rad Protein Assay Kit (Bio-Rad, California, USA).

### SLA-cell proliferation assay (SLA-CPA)

PBMC were isolated by density centrifugation through Ficoll-Hypaque (Rafer, Spain). The collected cells were cultured in RPMI 1640 supplemented with 10% heat-inactivated fetal bovine serum, 100 IU/ml penicillin, 100 μg/ml streptomycin, 2 mM L-glutamine, 50 μM 2-mercaptoethanol, and 1 mM sodium pyruvate. They were then plated in 96 well-plates and kept with RPMI 1640 medium alone (unstimulated) or with added SLA (10 μg/ml). All were kept in a humidified, 5% CO_2_ atmosphere at 37°C for 5 days. Cell proliferation was measured by bromodeoxyuridine incorporation using the Cell Proliferation Biotrak ELISA Kit (General Electric Healthcare Life Sciences, UK). The results are shown as a stimulation index (SI).

### Cytometric quantification of IFN-γ

IFN-γ production was determined in 50 μl of the supernatants from the above SLA-stimulated and control PBMC cultures using the BD Cytometric Bead Array Human Flex Set (Beckton Dickinson Biosciences, New Jersey, USA), as previously described (Carrillo et al., 2015). Supernatants were collected and stored at −20°C for cytokine analysis. Data were acquired using a FACSCalibur flow cytometer and analyzed using the Flow Cytometric Analysis Program Array (Beckton Dickinson Biosciences, New Jersey, USA). IFN-γ production was expressed (in pg/ml) as the difference between the concentration in SLA-stimulated and control supernatants.

### Statistical analysis

Statistical analyses were performed using the SPSS package (Chicago, IL, USA) or GraphPad Prism 7.0 software (GraphPad Software, CA, USA). Cut-offs were determined by calculating the area under the receiver operating characteristic (ROC) curve (AUC) and the 95% confidence interval (CI). Values for variables recorded before and after treatment were compared using the Mann–Whitney U-test. Significance was set at *p* < 0.05.

## Results and discussion

### The parasitological and serological tests identified cured VL, but not cured CL, LLL, or ML

The results of the PCR (in particular), rK39-ICT and IFAT tests for active and cured VL were significantly different, thus identifying the cured condition (Table 1).

**Table 1:** Percentage of positive results returned by parasitological, serological, and cellular immunity tests, in patients with active (A) and cured (C) leishmaniasis.

**Clinical condition**	**PCR (%)**		**rK39 (%)**		**IFAT (%)**		**SLA-CPA (%)**^**a**^		**IFN-**γ **(%)**^**b**^	
	**A**	**C**	**A**	**C**	**A**	**C**	**A**	**C**	**A**	**C**
Visceral leishmaniasis	67	3^***^	77	52^*^	90	56^*^	16	90^****^	14	84^****^
Cutaneous leishmaniasis	4	2	4	2	0	2	33	56	31	62
Leishmanial lymphoadenopathy	0	0	33	43	33	14	52	67	47	75
Mucosal leishmaniasis	0	0	100	67	50	67	33	100	33	100

a *The stimulation index cut-offs for SLA-CPA (ROC curve) were = 2.53 for VL, 2.26 for CL, 3.16 for LLL, and 4.23 for ML*.

b *The cut-offs for increased IFN-γ production after SLA stimulation of blood (ROC curve) were = 133.4 pg/ml for VL, 314.1 pg/ml for CL, 406.2 pg/ml for LLL, and 406.6 pg/ml for ML*.

* *p < 0.050*.

*** *p < 0.001*.

**** *p < 0.0001*.

In contrast, PCR detected parasite DNA in very few CL, LLL or ML blood samples with no significant differences recorded between the active and cured conditions. Similarly, the rk39-ICT and IFAT tests returned very similar results for the active and cured forms of these disease presentations (Table 1).

### Cell-mediated immunity: PBMC proliferation and IFN-γ production as biomarkers of cured VL

**Figure 1:**
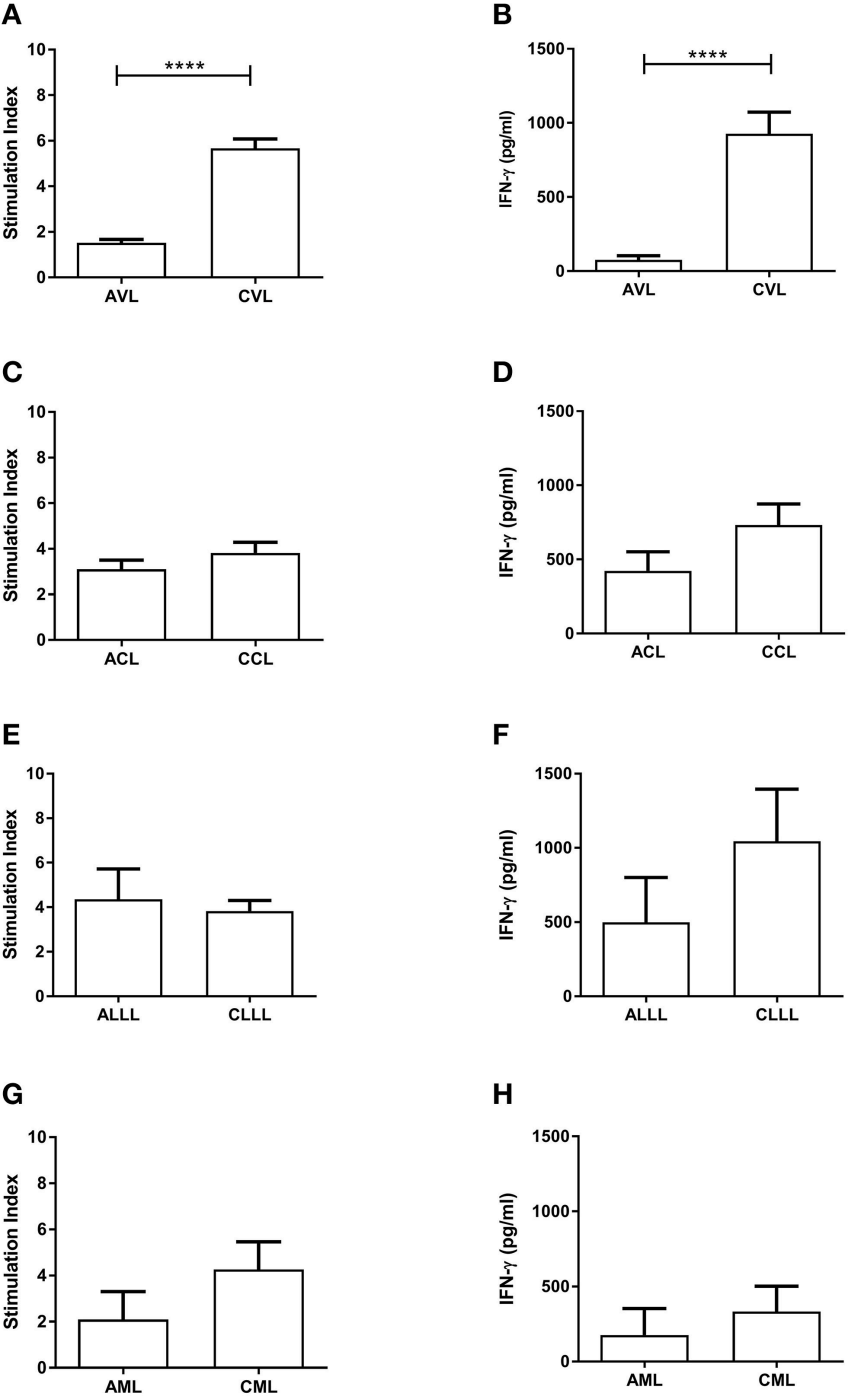
Proliferation of PBMC (SLA-CPA) **(A,C,E,G)** and associated IFN-γ production **(B,D,F,H)** from active and cured patients with different clinical forms of *L. infantum*-induced disease. ****p < 0.0001. AVL, Active Visceral Leishmaniasis; CVL, Cured Visceral Leishmaniasis; ACL, Active Cutaneous Leishmaniasis; CCL, Cured Cutaneous Leishmaniasis; ALLL, Active Leishmanial Localized Lymphadenopathy; CLLL, Cured Leishmanial Localized Lymphadenopathy; AML, Active Mucosal Leishmaniasis; CML, Cured Mucosal Leishmaniasis.

Analysis of cell-mediated immunity in the different clinical conditions revealed significant differences between active and cured conditions only for VL (Table 1): indeed, most of the patients with active VL showed no *Leishmania*-specific cell-mediated immunity, but did so after effective treatment, with PBMC proliferation confirmed (Figure 1A, *p* < 0.0001) and IFN-γ levels increased (Figure 1B, *p* < 0.0001) (cut-offs: Stimulation index = 2.53; IFN-γ = 133.4 pg/ml). No such conversion was seen in any other disease presentation type (Figures 1C–H).

### Lack of surrogate end-points to define cure in leishmaniasis

A confirmation of cure has traditionally been based on clinical features, such as the normalization of body temperature, a reduction in the size of the liver and spleen, and an increase in peripheral blood leukocytes, hemoglobin and platelets in VL, or the healing of lesions in CL. However, observations have to be made over long follow-up times since relapses may occur long after treatment ends. Unfortunately, PCR and serological tests are not good indicators of cure (WHO, 2017). PCR detects parasites in active VL, but after the first doses of treatment their numbers can fall dramatically (Castro et al., 2016) while clinical manifestations of disease remain. Further, the non-detection of peripheral blood parasites does not necessarily mean that the spleen, liver, or bone marrow are parasite-free, which might allow for the reactivation of clinical disease months later, as repeatedly reported in immunosuppressed patients (van Griensven et al., 2014). PCR results on their own cannot, therefore, provide a reliable marker of cure. Neither can serological tests (rK39-ICT and IFAT) be used to identify cure: antibodies are present in active VL but persist long after cure (Hailu et al., 2004; WHO, 2010).

### Cell-mediated immunity tests for monitoring treatment

The development of solid cell-mediated immunity rendering patients resistant to reinfection only occurs after successful treatment and the start of healing (Kemp et al., 1993; Kumar et al., 2014). Early monitoring of response to treatment (for both VL and CL) therefore requires specific biomarkers be identified that correlate with the development of this immunity (WHO, 2017). PBMC proliferation and increased IFN-γ production upon stimulation with SLA are expressions of this immunity, and can be easily and repeatedly evaluated *ex vivo* without patient sensitization (Carrillo et al., 2015). In addition, these two biomarkers have added value in terms of allowing a “no relapse” prognosis to be made: both are directly associated with the immunological control of the parasite and the absence of reactivation. By way of comparison, while PCR can confirm parasite elimination in blood, it cannot predict whether the disease might be reactivated by cryptic parasites in the target organs.

The appearance of cell-mediated immunity after successful treatment has also been reported for *Leishmania donovani* (WHO, 2017). PBMC proliferation and an associated increase in IFN-γ production upon the stimulation of blood samples with SLA might therefore provide global markers of cure in VL (Adem et al., 2016; Castro et al., 2016). Our group has already shown that a patent cell proliferative response remains long after treatment, that it is useful for monitoring disease in immunosuppressed patients with VL (Castro et al., 2016), and that it is associated with a lack of relapse (Hailu et al., 2004; Singh and Sundar, 2014).

### Lack of biomarkers for identifying cure in CL, LLL, and ML

The present results show that none of the tests examined could distinguish between active disease and cure in CL, LLL, or ML. In fact, many patients with CL and LLL returned positive SLA-CPA results (i.e., PBMC proliferation) *before* treatment, revealing an active cell-mediated immune response to be underway. The same response has been described in patients with active CL caused by *L. major*, which appears to be associated with the spontaneous healing of the lesion. In active CL, a lack of PBMC proliferation and low IFN-γ production (together with IL-4 production) following SLA challenge *in vitro* has been associated with severe disease and a lack of healing (Gaafar et al., 1995; Ajdary et al., 2000).

The mean stimulation index values recorded after SLA challenge shows the cell-mediated immune response against *Leishmania* to be strongest in patients cured of VL. Differences in this response between patients cured of VL and CL have been reported previously, and might reflect a stronger systemic Th-1 response to occur in the former given the need to clear a much heavier parasite burden (Turgay et al., 2010; Alimohammadian et al., 2012). The patients with LLL—perhaps an evolved form of CL—showed the strongest immunological response before treatment, with *Leishmania* parasites present in affected lymph glands near the site of the *Phlebotomus* bite (Horrillo et al., 2015). The intense immunological activity in lymph nodes probably results in an immune over-reaction against the parasite. No information on the immunological features of LLL has previously been reported.

The present study suffers from the limitation of the imbalance in patient numbers between the groups; indeed, the numbers of patients with active ML and LLL are too small to be able to draw safe conclusions. To our knowledge, however, this is the first time that the immune response of such a large number of individuals with different clinical forms of disease caused by *L. infantum*, and all from the same endemic area, has been examined.

Individual differences in the immune response to *L. infantum* result in different clinical outcomes. The absence of a specific cell-mediated response to the parasite in patients with active VL allows *in vitro* SLA-activated PBMC proliferation and the associated increase in IFN-γ production—all of which occurs after successful treatment—to act as biomarkers of cure in this disease type. Their use might improve patient follow-up and reduce the cost of clinical trials of VL treatments. In patients with active CL, LLL, and ML, the cell-mediated response observed in a large number of patients seems capable of preventing the spread of the parasite to the internal organs, but it provides no indicator of cure since there are no differences before and after treatment. Biomarkers of cure of CL, LLL, and ML are, therefore, still needed.

## Ethics statement

This study was approved by the Hospital de Fuenlabrada (Madrid) Ethics and Research Committee (APR 12-65 and APR 14-64). All patients gave their written informed consent to be involved.

## Author contributions

JS, AR-M, JR-G, EC, and JM contributed conception and design of the study. BM, AR-M, AC, LM, MA, and LH recruited patients and collected samples. LB, LF, AI-M, CS, CC, JN, and SO did immunological tests. LB, BM, and JS organized the database and performed the statistical analysis. LB and BM wrote the first draft of the manuscript. JS, EC, and JM wrote sections of the manuscript. All authors contributed to manuscript revision, read, and approved the submitted version.

### Conflict of interest statement

The authors declare that the research was conducted in the absence of any commercial or financial relationships that could be construed as a potential conflict of interest.
